# C-reactive protein levels and cognitive functions in patients with bipolar disorder

**DOI:** 10.1192/j.eurpsy.2023.1482

**Published:** 2023-07-19

**Authors:** O. Elleuch, M. Maalej, W. Guidara, M. Naifar, M. Maalej, F. Ayadi

**Affiliations:** 1 Department of psychiatry C, Hedi Chaker University Hospital of Sfax; 2 Laboratory of Research “Molecular Basis of HumanDiseases”, LR19ES13, Faculty of Medicine, University of Sfax; 3 Laboratory of Biochemistry, Faculty of Medicine of Sfax & Habib Bourguiba Hospital, Sfax, Tunisia

## Abstract

**Introduction:**

The pathophysiology of bipolar disorder (BD) is complex and remains uncertain to this day.

Several hypotheses have been suggested, including the involvement of inflammatory mechanisms in its pathogenesis and in eventual cognitive impairment. C-reactive protein (CRP) is one of the most commonly used inflammatory markers. High-sensitivity CRP (hs- CRP) is a more sensitive marker.

**Objectives:**

We aimed to examine hs-CRP levels in patients with BD, and to investigate its relationship with cognitive functions.

**Methods:**

We conducted a cross-sectional study between June 2016 and July 2018 on drug-free BD patients. These participants were hospitalized at the “C” Psychiatry department of HediChaker University Hospital in Sfax-Tunisia which accepts only male patients. The diagnosis of BP disorder was established according to DSM-5 criteria.

We used the Montreal Cognitive Assessment (MoCA) scale to assess cognitive functions in our patients, and blood samples were collected to analyze hs-CRP levels.

**Results:**

Our study included 33 patients whose median age was 33 years, with an interquartile range of 27.5-44 years. The majority (90.3%) were diagnosed with type I of bipolar disorder and 9.7% were diagnosed with type II. At the time of the study, 82.4% had a maniac episode and 17.6% had a depressive one.

The median MOCA scale score was 24 with an interquartile range of 19-26, and the analysis of hs-CRP values revealed a median level of 2.1 (interquartile range: 1.2-7.3).

There was no significant correlation between hs-CRP levels and MOCA scores nor its domains. Table 1 shows the results of this correlation analysis.

**Table1:** correlation results between hs-CRP levels and MOCA domains

		MOCA	Visuospatial and executive functions	naming	attention	language	abstraction	memory	orientation
hs-CRP	r=	0.157	0.031	-0.092	-0.020	0.099	0.071	0.055	-0.151
	p=	0.415	0.873	0.636	0.918	0.610	0.714	0.775	0.435

We also did not find any significant difference in hs-CRP levels between patients with depressive and maniac episodes.

**Conclusions:**

Our study showed no significant relationship between inflammation and cognitive impairment in bipolar disorder. Further research is needed to better investigate the role of inflammatory processes in this disorder.

**Disclosure of Interest:**

None Declared

